# Gastrocnemius muscle properties in children with cerebral palsy: An exploratory cross‐sectional study of variability in gastrocnemius properties

**DOI:** 10.1113/EP093772

**Published:** 2026-09-28

**Authors:** Elizabeth R. Boyer, Christiana J. Raymond‐Pope, Daniel B. Hoffman, Marshall H. Taniguchi, Tom F. Novacheck, Braydon A. Crum, Peter D. Kim, Sarah M. Greising

**Affiliations:** ^1^ Research Department Gillette Children's St. Paul Minnesota USA; ^2^ Orthopedic Surgery Department University of Minnesota Minneapolis Minnesota USA; ^3^ School of Kinesiology University of Minnesota Minneapolis Minnesota USA

**Keywords:** botulinum toxin, cerebral palsy, microbiopsy, muscle properties, needle biopsy, selective dorsal rhizotomy, spasticity

## Abstract

Muscle strength is a key predictor of physical functioning, yet the influence of patient and treatment factors on muscle properties in young people with cerebral palsy is not fully known. We explored whether medial gastrocnemius properties vary by age, ambulatory status and spasticity treatment (botulinum toxin or selective dorsal rhizotomy). In this exploratory cross‐sectional study, microbiopsies of the medial gastrocnemius were obtained from 16 children (6–17 years of age) with cerebral palsy. Outcomes included muscle thickness, collagen content, myofibre cross‐sectional area, capillaries, myofibres with centrally located nuclei, lipid area, satellite cell density and myosin heavy chain myofibre type. Myofibre cross‐sectional area (*P* = 0.02) and myofibres with centrally located nuclei (*P* = 0.02) were positively correlated with age. Non‐ambulatory individuals had smaller myofibre cross‐sectional area (*P* = 0.02). The number of botulinum toxin injections was associated with more capillary contacts per myofibre (*P* = 0.04). Selective dorsal rhizotomy was not associated with any statistically significant findings. Preliminary data suggest that gastrocnemius muscle properties might vary across patient factors and spasticity treatment history. Confirmatory prospective studies with matched control groups are needed to evaluate properly the effects of age, ambulatory status, botulinum toxin and selective dorsal rhizotomy.

## Abstract (highlights)

- **What is the central question of this study?**
  Do gastrocnemius properties vary by age, ambulation, botulinum toxin or selective dorsal rhizotomy in cerebral palsy?
- **What is the main finding and its importance?**
  Hypothesis‐generating results suggest that gastrocnemius properties might vary by demographics and treatment. Myofibre cross‐sectional area and the number of myofibres with centrally located nuclei increased with age. Ambulatory individuals had larger myofibre cross‐sectional area than non‐ambulatory individuals. The number of botulinum toxin injections was positively correlated with capillary contacts per myofibre. Confirmatory studies are needed to verify these findings.

## 1 INTRODUCTION

The neurological brain difference or injury that causes cerebral palsy (CP) leads to impaired motor control, balance, strength, muscle tone and muscle quality (Dan et al., 2025; Graham et al., 2016). These impairments impact physical function and are generally more severe as gross motor classification system (GMFCS) level increases (Palisano et al., 1997). Some evidence suggests higher GMFCS levels are associated with poorer muscle quality (Johnson et al., 2009; Noble et al., 2014; Pitcher et al., 2015; Schless et al., 2019), although there are limited data from non‐ambulatory individuals (Johnson et al., 2009; Kahn et al., 2023; Meza et al., 2026; Pitcher et al., 2015).

The co‐occurrence of multiple impairments complicates identification of the impairment(s) most responsible for reduced gross motor function. Multivariate models consistently identify muscle strength as a major contributor (Eek & Beckung, 2008; Holmes et al., 2018; Kim & Park, 2011; Ross & Engsberg, 2007; Schwartz et al., 2022). Given that individuals with CP exhibit weakness (Barrett & Lichtwark, 2010; Eek & Beckung, 2008), interventions that preserve or enhance muscle quality and strength should be prioritized. At the same time, spasticity can affect function and comfort and is treatable with botulinum toxin (BoNT‐A) or selective dorsal rhizotomy (SDR).

The scientific understanding of how BoNT‐A and SDR affect skeletal muscle properties in CP is still nascent, particularly for SDR, although the negative effects of BoNT‐A on muscle size and growth at 1–12 months post‐injection have been shown repeatedly in prospective CP studies (Alexander et al., 2018; De Beukelaer et al., 2022; Deschrevel et al., 2024; Lambrechts et al., 2025; Peeters et al., 2022; Williams et al., 2013). Although absolute muscle size measured by ultrasound or MRI may increase in children after BoNT‐A (Barber et al., 2013), normalized muscle size and growth rate decrease compared with baseline (Barber et al., 2013; Williams et al., 2013) or with age‐ and severity‐matched controls (growth rate BoNT‐A, −30% vs. no BoNT‐A: +32%) (Lambrechts et al., 2025). To our knowledge, the only longitudinal study at the myofibre level to measure myofibre cross‐sectional area (CSA) found a decrease in normalized CSA at 3 months but an increase at 6 months (Deschrevel et al., 2024). The trajectory of muscle growth in the months following SDR is incompletely known. Normalized whole‐muscle size measured via ultrasound at 12 months post‐SDR was similar to pre‐SDR, and growth was comparable to a no‐SDR group (Verreydt et al., 2025). Regarding other muscle properties, less is known about whether or how BoNT‐A and SDR affect the muscles of children with CP. Collagen and fat (estimated via ultrasound echo‐intensity) increased ∼2–3 months post‐BoNT‐A (Lambrechts et al., 2025; Peeters et al., 2022), then returned to baseline at 6 months (Lambrechts et al., 2025).

Muscle strength depends on contractile and biochemical properties, not only size, hence measuring these properties could help in understanding the effects of BoNT‐A and SDR treatment. Such longitudinal treatment data do not exist for children with CP who received SDR, although a case report found that gastrocnemius properties such as collagen, lipids, capillaries and satellite cells were not drastically different compared with two children who did not receive SDR (Raymond‐Pope et al., 2023). A recent BoNT‐A biopsy study in 12 toxin‐naive, ambulatory children found that myofibre CSA decreased at 3 months, then increased at 6 months relative to baseline; the proportion of myosin heavy chain (MyHC) slow expressing type I myofibres increased and remained elevated, whereas type IIx myofibres did the opposite; capillary contact per myofibre decreased but returned to baseline by 6 months; and the number of satellite cells increased slightly, albeit not significantly (Deschrevel et al., 2024). Despite many strengths of the study design, the absence of a no‐BoNT‐A CP control group limited the interpretation of natural history in CP and the dose effects of repeated BoNT‐A.

In this exploratory hypothesis‐generating study, we aimed to expand the understanding of muscle properties across all GMFCS levels and two common spasticity treatments. Specifically, we explored potential differences in medial gastrocnemius muscle properties by age, ambulatory status, prior BoNT‑A and prior SDR versus no SDR.

## 2 MATERIALS AND METHODS

### 2.1 Ethical approval

Written informed consent and assent (for those ≥8 years who had cognitive capacity to assent) were obtained prior to participation. The study conformed to the standards set by the *Declaration of Helsinki*. The study procedures were approved by the local ethics committee (University of Minnesota Institutional Review Board, STUDY00018134).

This exploratory cross‐sectional study was not powered for hypothesis testing; rather, analyses were conducted to estimate effect sizes and variability to inform future studies. A convenience sample of children (<18 years of age) with spastic or mixed‐tone CP undergoing clinical procedures using general anaesthesia was recruited between May 2023 and September 2024. Individuals were excluded if they did not read or speak English. Demographics and treatment history were extracted from the medical record.

### 2.2 Ultrasound

Ultrasound was used to measure medial gastrocnemius muscle thickness. All ultrasound images were obtained using the same machine (Philips EPIQ 7, manufacture date 2021, Amsterdam, The Netherlands) by one of two trained ultrasound technicians before the procedure requiring general anaesthesia. The participant was positioned prone, with the knee flexed 25° (Cenni et al., 2018; Schless et al., 2019). The ankle was in resting plantarflexion (Cenni et al., 2018; Obst et al., 2017; Pitcher et al., 2015; Schless et al., 2019). Hip‐flexion contractures were accommodated with pillows or rolled towels. Knee‐flexion contractures prevented achievement of 25° in two participants (30° and 44°). An 18‐4 linear MHz transducer was used along with the following settings: gain 68%, dynamic range 68 dB, depth 4.5 cm (adjusted based on size) and focus 3 cm. Care was taken to minimize pressure from the ultrasound transducer to avoid muscle compression. The ultrasound technician scanned in the transverse plane at one‐third of the distance between the popliteal fossa and intermalleolar line (O'Brien T et al., 2017) to visualize the probe location corresponding to maximal medial gastrocnemius thickness. At least three images were obtained. Muscle thickness was quantified by a single investigator using FIJI (Schneider et al., 2012) as the thickest part between the superficial and deep aponeuroses (May et al., 2021). The average of the three images was reported.

The side for both ultrasound and biopsy was indicated as: (1) the hemiplegic or more‐involved limb noted in physician documentation; or (2) the side with a lower selective motor control score averaged across the lower extremity muscles; or (3) a random side if there was no/minimal difference based on selectivity.

### 2.3 Muscle biopsy and processing

The ultrasound‐guided medial gastrocnemius microbiopsy was collected either before or after the clinical procedure, according to the preference of the physician. Eight physicians obtained samples from 16 participants. A 16 gauge, 10 cm disposable core biopsy instrument (Bard, Semi‐Automatic 1610MS, Franklin Lakes, NJ, USA) was used to obtain the microbiopsy. A sterile preparation was used. Participants were most often supine, with the hip externally rotated and knee flexed. Physicians visually identified the greatest medial gastrocnemius thickness and sampled medial to lateral. An in‐plane ultrasound approach was used to visualize the shaft of the needle and tip as it entered the muscle to avoid injury to the nerve or vessels and determine containment within the medial gastrocnemius prior to sampling. Three samples were obtained through the same percutaneous insertion (and up to four were obtained if quality was in question). Each of the muscle samples obtained weighed ∼10 mg. For each participant, two high‐quality samples were saved for histological processing and the other for biochemical processing of total collagen content by hydroxyproline. Harvested tissue was saved and processed based on published methods (Raymond‐Pope et al., 2023). Briefly, histological sections were placed in a mould containing optimal cutting temperature compound (OTC) and frozen in frozen in 2‐methylbutane cooled by liquid nitrogen, then saved at −80°C until processing. Muscles saved for biochemical analysis were flash frozen in liquid nitrogen, then saved at −80°C until processing.

Muscles were sectioned serially at 10 µm thickness and stained with various methods, including Haematoxylin and Eosin, Masson's Trichrome, alkaline phosphatase (capillary contact per myofibre), Oil Red O (lipids); and the following primary antibodies: laminin (ab11575, 5 µg/ml), nuclei (DAPI, Thermo Fisher Scientific catalogue no. D21490; 10 µg/ml) and Pax7 (satellite cells; 5 µg/ml), and MyHC isoforms slow (MyHCslow; 5 µg/ml) and 2a (BA‐D5; MyHC2A; 5 µg/ml, SC‐71) acquired from the Developmental Hybridoma Studies Bank (Iowa City, IA, USA). Corresponding host‐ and isotype‐specific fluorescently conjugated secondary antibodies were used. Individual myofibres were classified as type I or IIa based on MyHCslow or MyHC2A expression, respectively. Myofibres without staining were classified as type IIx; co‐expression was identified when multiple stains were apparent (e.g., I/IIa). Imaging was conducted using a ×20 objective (0.75 NA, 0.5 µm/pixel resolution) on the TissueScope LE slide scanner (Huron Digital Pathology) or a Nikon Ti2 laser scanning microscope using a Plan APO ×20 (0.8 NA) or Plan APO ×60 (1.42 NA) objective. In all cases, the entire muscle section was analysed, and the large image acquisition add‐on within Nikon NIS‐Elements AR automated the stitching of smaller images to capture images of the whole muscle section. During all processing and analysis, investigators were blinded to group.

### 2.4 Statistical analysis

Statistical analysis was conducted in R (v.4.4.2). Claude Sonnet v.4.6 was used to aid in statistical analyses and R coding, which was verified by the first author. The five‐number statistical summary (minimum, first quartile, median, third quartile and maximum) was provided for this hypothesis‐generative study to illustrate cohort and subgroup distributions and overlap. Pairwise deletion was used for missingness. Data were assessed for normality. For categorical independent variables (ambulatory status, prior SDR [yes, no]), main effects were assessed using a non‐parametric Mann–Whitney *U*‐test, which was used to calculate rank‐based rank biserial correlations (*r*_rb_) effect sizes (Fiel Peres, 2026). The 95% confidence intervals (95% CIs) were estimated via a percentile bootstrap (1000 resamples). Spearman correlations (*r*_s_) were conducted for continuous independent variables (age, number of prior BoNT‐A injections), with 95% CIs calculated using the Fisher *z*‐transformation (Bishara & Hittner, 2017). Statistical significance was determined based on *P *< 0.05.

## 3 RESULTS

Among the 57 eligible participants contacted, 16 (28%) participated in this exploratory study (Table 1). The SDR group was 3–10 years post‐SDR, and many had multiple treatments (Table 2; Table A1). The BoNT‐A group was 1–10 years post‐BoNT‐A.

**TABLE 1:** Demographics of participants at enrolment.

Characteristic	All	Ambulatory	Non‐ambulatory	No BoNT‐A to gastrocnemius	BoNT‐A to gastrocnemius	No SDR (ambulant only)	SDR (ambulant only)
All, *n*	16	10	6	7	9	7	3
Age, years, mean (SD) [range]	11(4) [6–16]	12(4) [7–16]	9(3) [6–13]	11(4) [6–16]	11(4) [7–16]	13(4) [7–16]	11(4) [8–15]
Sex, *n* (%)							
Female	7 (44)	5 (50)	2 (33)	3 (43)	4 (44)	4 (57)	1 (33)
Male	9 (56)	5 (50)	4 (66)	4 (57)	5 (55)	3 (43)	2 (66)
GMFCS level, *n* (%)							
I	1 (6)	1 (10)	0	0	1 (11)	1 (14)	0
II	6 (38)	6 (60)	0	2 (29)	4 (44)	4 (57)	2 (66)
III	3 (19)	3 (30)	0	2 (29)	1 (11)	2 (29)	1 (33)
IV	2 (13)	0	2 (33)	0	2 (22)	0	0
V	4 (25)	0	4 (66)	3 (43)	1 (11)	0	0
Topography, *n* (%)							
Hemiplegia	2 (13)	2 (20)	0 (0)	0 (0)	2 (22)	2 (29)	0 (0)
Diplegia	5 (31)	5 (50)	0 (0)	3 (43)	2 (22)	2 (29)	3 (100)
Tri‐ or quadriplegia	9 (56)	3 (30)	6 (100)	4 (57)	5 (56)	3 (43)	0 (0)
Predominant tone type, *n* (%)							
Spastic	12 (75)	9 (90)	3 (50)	6 (86)	6 (67)	6 (86)	3 (100)
Mixed	4 (25)	1 (10)	3 (50)	1 (14)	3 (33)	1 (14)	0 (0)

Abbreviations: BoNT‐A, botulinum toxin; GFMCS, gross motor function classification system; SDR, selective dorsal rhizotomy.

**TABLE 2:** Participant treatment history^†^ related to the biopsied gastrocnemius.

Patient ID	Age at biopsy	GMFCS level	Study group(s)	Number of prior BoNT‐A gastroc	Age at prior BoNT‐A gastroc	Time since last BoNT‐A gastroc	Age at prior SDR	SDR details
1	12 y 1 mo	V	Non‐ambulatory	0	N/A	N/A	N/A	N/A
2	7 y 11 mo	II	Ambulatory, no SDR	5	Five, from 3 y 11 mo to 6 y 4 mo^†^	1 y 6 month	N/A	N/A
3	11 y 1 mo	II	Ambulatory, SDR	0	N/A	N/A	6 y 0 month	L2–S1, cauda
4	16 y 7 mo	III	Ambulatory, no SDR	0	N/A	N/A	N/A	N/A
5	7 y 10 mo	V	Non‐ambulatory	2	6 y 6 mo, 7 y 0 mo	0 y 10 mo	N/A	N/A
6	13 y 1 mo	II	Ambulatory, no SDR	1	4 y 6 mo	8 y 7 mo	N/A	N/A
7	16 y 7 mo	I	Ambulatory, no SDR	17	17, from 3 y 0 mo to 13 y 8 mo^†^	2 y 11 mo	N/A	N/A
8	16 y 4 mo	II	Ambulatory, no SDR	0	N/A	N/A	N/A	N/A
9	15 y 10 mo	II	Ambulatory, no SDR	9	Nine, from 11 y 7 mo to 14 y 5 mo^†^	1 y 5 mo	N/A	N/A
10	8 y 8 mo	II	Ambulatory, SDR	1	4 y 4 mo	4 y 5 mo	5 y 10 mo	L1–S2 cauda
11	10 y 5 mo	V	Non‐ambulatory	0	N/A	N/A	N/A	N/A
12	15 y 2 mo	III	Ambulatory, SDR	1	4 y 11 mo	10 y 3 mo	5 y 8 mo	L1–S2 conus
13	8 y 4 mo	III	Ambulatory, no SDR	0	N/A	N/A	N/A	N/A
14	6 y 10 mo	V	Non‐ambulatory	0	N/A	N/A	N/A	N/A
15	7 y 1 mo	IV	Non‐ambulatory	4	5 y 0 mo, 5 y 2 mo, 5 y 8 mo, 6 y 0 mo	1 y 1 mo	N/A	N/A
16	13 y 1 mo	IV	Non‐ambulatory	2	8 y 1 mo, 9 y 4 mo	3 y 10 mo	N/A	N/A

Abbreviations: BoNT‐A, botulinum toxin; gastroc, gastrocnemius; GMFCS, gross motor function classification system; L, lumbar; mo, months; N/A, not applicable; S, sacral; SDR, selective dorsal rhizotomy; SEMLS, single event multilevel surgery; y, years.

^†^ Detailed age at treatment is provided in Table A1.

Minor side effects of biopsy were reported in four participants (25%); two reported mild pain (2/10 on Faces Pain Scale), one reported swelling and one reported discoloration/bruising. Physicians reported no intraoperative subdermal haematoma or other side effects.

Sufficient biopsy amount and quality were obtained for nearly all participants, except participant 1, for whom only muscle thickness and collagen were measured (Tables 3 and 4). The average number of myofibres per sample was 1134, with a range from 298 to 3475 myofibres across all samples processed and evaluated. Histological staining from a representative ambulatory participant is shown in Figure 1.

**TABLE 3:** Spearman correlation coefficients (95% confidence interval) for age and number of botulinum toxin injections to the biopsied gastrocnemius.

Parameter	*n*	Age	Number of botulinum toxin injections
Gastrocnemius muscle thickness	9	0.46 (−0.29, 0.86) *P* = 0.21	−0.13 (−0.73, 0.58) *P* = 0.73
Myofibre cross‐sectional area	13	0.65 (0.16, 0.86) *P* = 0.02	0.06 (−0.51, 0.59) *P* = 0.84
Collagen content, µg/mg	13	0.17 (−0.42, 0.66) *P* = 0.58	0.41 (−0.18, 0.78) *P* = 0.16
Capillaries per myofibre, average	15	0.08 (−0.45, 0.57) *P* = 0.79	0.53 (0.02, 0.82) *P* = 0.04
Percentage of myofibres with centrally located nuclei	15	0.61 (0.14, 0.85) *P* = 0.02	0.25 (−0.30, 0.67) *P* = 0.37
Lipid droplet area	15	0.38 (−0.16, 0.75) *P* = 0.16	0.39 (−0.15, 0.75) *P* = 0.15
Satellite cells per 100 myofibres	15	0.32 (−0.23, 0.72) *P* = 0.24	0.34 (−0.21, 0.73) *P* = 0.22
Myofibre type proportion			
I	14	−0.41 (−0.77, 0.16) *P* = 0.15	0.02 (−0.51, 0.55) *P* = 0.93
I/IIa (co‐expressing)	14	0.07 (−0.48, 0.58) *P* = 0.82	0.34 (−0.23, 0.74) *P* = 0.24
IIa	14	0.38 (−0.18, 0.76) *P* = 0.18	0.23 (−0.33, 0.68) *P* = 0.42
IIa/IIx (co‐expressing)	14	0.06 (−0.49, 0.57) *P* = 0.85	−0.52 (−0.82, 0.01) *P* = 0.06
IIx	14	0.28 (−0.29, 0.71) *P* = 0.33	−0.05 (−0.56, 0.50) *P* = 0.88

**TABLE 4:** Outcome values for each group and between‐group statistical differences^†^.

Parameter	All	Ambulatory	Non‐ambulatory	Between‐group comparison on ambulatory status *r*_rb_ (95% CI) *U*‐statistic, *P*‐value	No SDR	SDR	Between‐group comparison on SDR *r*_rb_ (95%CI) *U*‐statistic, *P*‐value
**Muscle thickness, cm **							
*n*	10	7	2	*r*_rb _= 1.00 (1.00,1.00)	4	3	*r*_rb_ = −0.33 (−1.00,0.71)
Median (25th percentile, 75th percentile) [min–max]	1.15 (0.94,1.27) [0.46–1.95]	1.16 (1.08,1.25) [0.91–1.41]	0.64 [0.46, 0.81]	*U* = 14, *P* = 0.06	1.08 (0.99,1.22) [0.91–1.41]	1.20 [1.15, 1.20, 1.29]	*U* = 4, *P* = 0.63
**Myofibre cross‐sectional area, µm^2^**							
*n*	14	9	4	*r*_rb _= 0.89 (0.49,1.00)	6	3	*r*_rb _= −0.22 (−1.00,0.69)
Median (25th percentile, 75th percentile) [min–max]	2237 (1363,4594) [927–8522]	2793 (2050,4792) [1198–5641]	1212 (1076,1363) [927–1562]	*U* = 34, *P* = 0.02	3026 (1888,4594) [1198‐5641]	2793 [2424, 2793, 5082]	*U* = 7, *P* = 0.71
**Collagen, µg/mg **							
*n*	14	7	6	*r*_rb _= −0.52 (−1.00, 0.17)	5	2	*r*_rb _= 1.00 (1.00,1.00)
Median (25th percentile, 75th percentile) [min–max]	9.9 (6.7,16.3) [1.4–31.0]	8.9 (6.9,12.6) [1.4‐16.4]	17.0 (11.8,21.7) [4.8–31.0]	*U* = 10, *P* = 0.13	9.0 (8.9,16.1) [7.3‐16.4]	4.0 [1.4, 6.5]	*U* = 10, *P* = 0.08
**Capillaries per myofibre, average **							
*n*	16	10	5	*r*_rb _= 0.00 (−0.72, 0.67)	7	3	*r*_rb _= 0.62 (0.00,1.00)
Median (25th percentile, 75th percentile) [min–max]	0.25 (0.17,0.32) [0.06–0.44]	0.25 (0.15,0.32) [0.06–0.44]	0.20 (0.18,0.32) [0.14–0.35]	*U* = 25, *P* = 1.00	0.31 (0.23,0.36) [0.06–0.44]	0.14 [0.10, 0.14, 0.24]	*U* = 17, *P* = 0.18
**Myofibre with centrally located nuclei, %**							
*n*	16	10	5	*r*_rb _= 0.42 (−0.24, 0.94)	7	3	*r*_rb_ = −0.14 (−1.00,0.67)
Median (25th percentile, 75th percentile) [min–max]	4 (3, 14) [2–34]	5 (3,16) [2–34]	3 (3,5) [3–6]	*U* = 35.5, *P* = 0.22	4 (3,20) [2–34]	5 [4, 5, 13]	*U* = 9, *P* = 0.82
**Lipid droplets, % per muscle area**							
*n*	16	10	5	*r*_rb _= 0.48 (−0.20, 1.00)	7	3	*r*_rb _= −0.14 (−1.00,0.75)
Median (25th percentile, 75th percentile) [min–max]	2.5 (1.7,3.8) [0.5–10.4]	3.0 (2.5,4.5) [0.6–10.4]	2.0 (0.9,2.4) [0.5–5.8]	*U* = 37, *P* = 0.16	3.0 (2.2,4.1) [0.6–10.4]	3.0 [2.5, 3.0, 6.0]	*U* = 9, *P* = 0.82
**Satellite cells per 100 myofibres **							
*n*	16	10	5	*r*_rb _= 0.64 (0.00, 1.00)	7	3	*r*_rb_ = 0.33 (−0.50,1.00)
Median (25th percentile, 75th percentile) [min–max]	14 (9,23) [6–62]	17 (13,28) [6–62]	9 (6,13) [6–14]	*U* = 41, *P* = 0.06	18 (14,31) [6–62]	13 [10, 13, 20]	*U* = 14, *P* = 0.49
**Myofibre type I, %**							
*n*	15	9	5	*r*_rb_ = −0.51 (−1.00, 0.14)	6	3	*r*_rb _= −0.44 (−1.00,0.33)
median (25%tile, 75%tile) [min‐max]	45 (30,60) [5–70]	37 (22,58) [5—64]	61 (42,67) [25–70]	*U* = 11, *P* = 0.14	29 (20,53) [5–64]	45 [37, 45, 60]	*U* = 5, *P* = 0.37
**Myofibre type I/IIa, %**							
*n*	15	9	5	*r*_rb _= 0.33 (−0.44, 0.93)	6	3	*r*_rb _= 0.56 (−0.33,1.00)
Median (25th percentile, 75th percentile) [min–max]	5 (3,12) [0–41]	11 (4,14) [2–41]	5 (3,9) [0–14]	*U* = 30, *P* = 0.35	12 (7,16) [7–41]	4 [2, 4, 11]	*U* = 14, *P* = 0.25
**Myofibre type IIa, %**							
*n*	15	9	5	*r*_rb _= 0.29 (−0.50,1.00)	6	3	*r*_rb _= 0.89 (0.43,1.00)
Median (25th percentile, 75th percentile) [min–max]	25 (20,36) [13–46]	28 (22,43) [17–45]	25 (13,26) [13–46]	*U* = 29, *P* = 0.42	36 (29,43) [22–45]	18 [17, 18, 25]	*U* = 17, *P* = 0.05
**Myofibre type IIa/IIx, %**							
*n*	15	9	5	*r*_rb_ = 0.20 (−0.60,0.86)	6	3	*r*_rb_ = −0.56 (−1.00,0.65)
Median (25th percentile, 75th percentile) [min–max]	18 (8,24) [3–36]	18 (10,25) [3–35]	9 (8,20) [3–36]	*U* = 27, *P* = 0.59	15 (8,20) [3–25]	25 [10, 25, 35]	*U* = 4, *P* = 0.25
**Myofibre type IIx, %**							
*n*	15	9	5	*r*_rb _= 0.24 (−0.51,0.96)	6	3	*r*_rb _= 0.22 (−1.00,1.00)
Median (25th percentile, 75th percentile) [min–max]	0.4 (0.3,2.3) [0.2–9.9]	0.4 (0.3,1.6) [0.2–9.9]	0.3 (0.2,0.6) [0.2–6.0]	*U* = 28, *P* = 0.50	0.4 (0.4,1.3) [0.3–4.2]	0.3 [0.2,0.3, 9.9]	*U* = 11, *P* = 0.70

*Note*: Between‐group comparisons are presented as effect sizes (i.e., rank biserial correlation coefficients, *r*_rb_) and *P*‐values.

^†^ If there were only two or three observations, all observations are listed in square brackets.

Abbreviations: BoNT‐A, botulinum toxin; GFMCS, gross motor function classification system; *r*_rb_, rank biserial correlation coefficients; SDR, selective dorsal rhizotomy.

**FIGURE 1:**
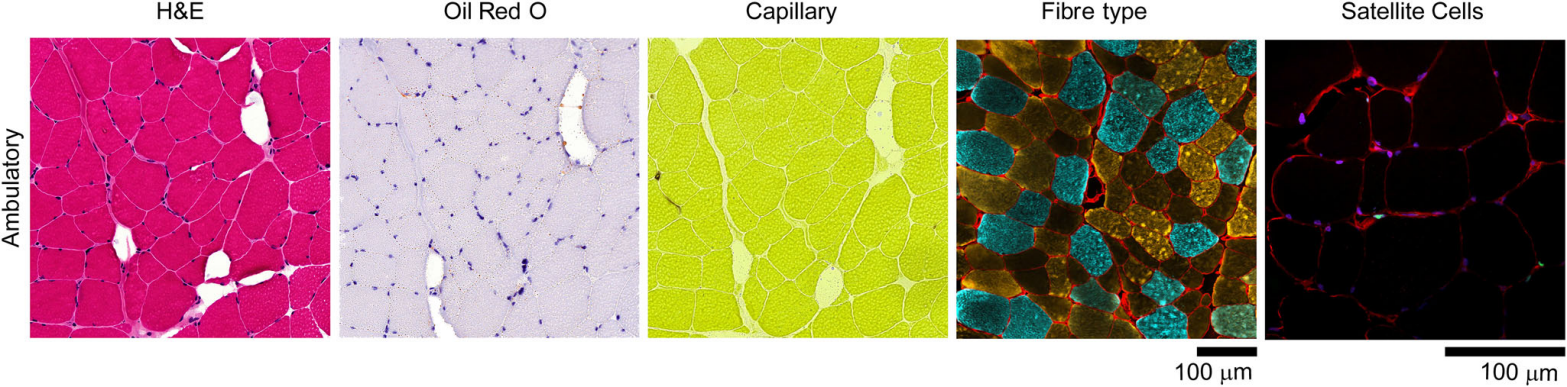
Representative histological images from an ambulatory patient. H&E images were used to evaluate muscle qualitatively and to quantify myofibres with centrally located nuclei; Oil Red O staining was used to quantity lipid droplets; capillaries; myofibre type (red, laminin; yellow, type IIa; teal, type I; black, type IIx; green, co‐expressing I/IIa myofibres); and satellite cells (red, laminin; green, Pax7^+^ satellite cell; blue, nuclei). Images are with scale bars for the images represent 10 µm, except for the satellite cell image (100 µm). Abbreviations: H&E, Haematoxylin and Eosin.

### 3.1 Age

Myofibre CSA and myofibres with centrally located nuclei were positively correlated with age (both *P* = 0.02; Table 3). No other outcomes were statistically significant for age.

### 3.2 Ambulatory status

Myofibre CSA was statistically smaller in non‐ambulatory compared with ambulatory individuals (*P* = 0.02; Table 4 and Figure 2). No other outcomes were statistically significant for ambulatory status, although muscle thickness (*r*_rb_ = 1.00, *P* = 0.06) and satellite cells (*r*_rb_ = 0.64, *P* = 0.06) tended to be greater in ambulatory versus non‐ambulatory individuals.

**FIGURE 2:**
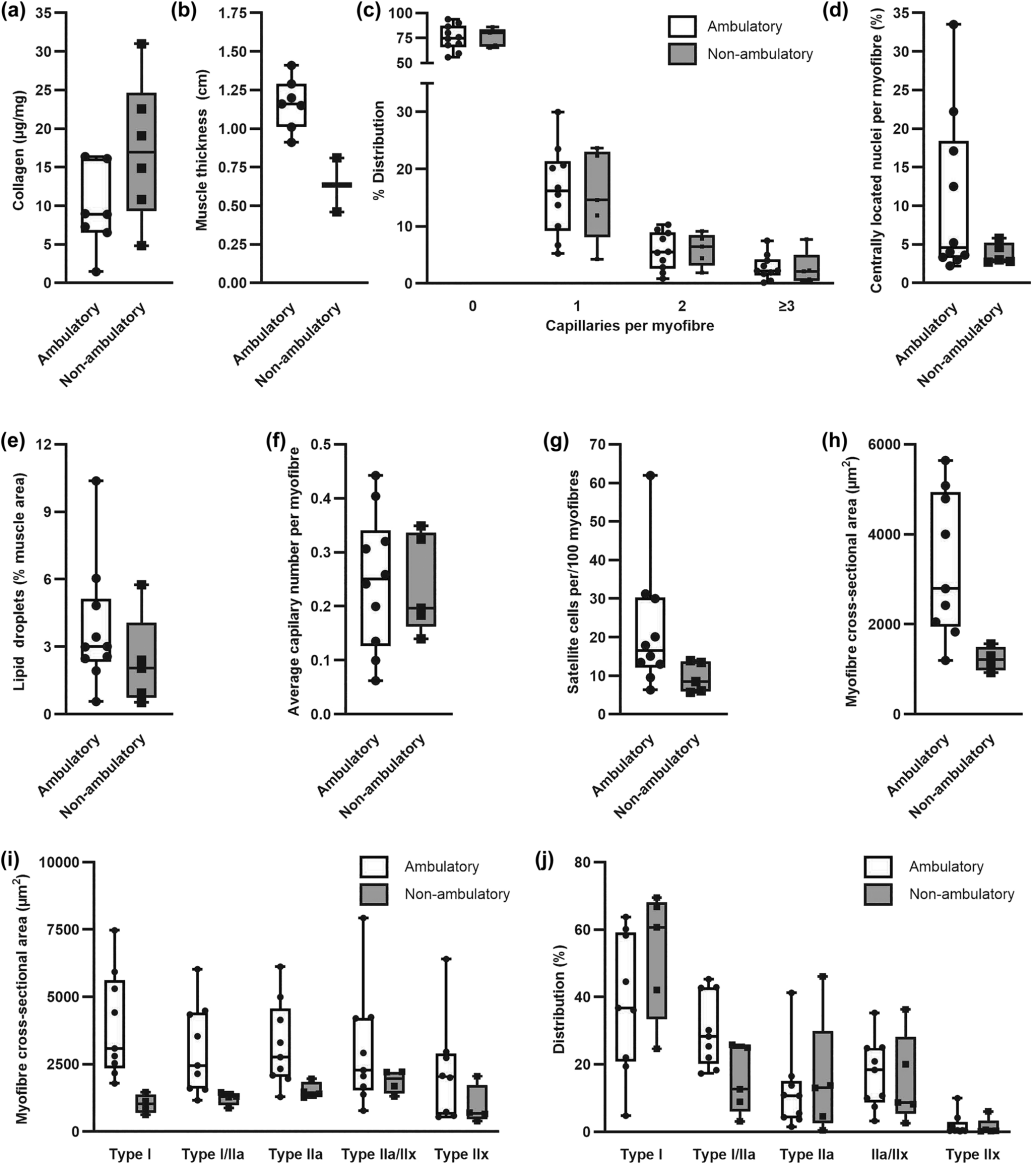
Graphical depiction of all quantitative analyses in box‐and‐whisker plots categorized by ambulatory and non‐ambulatory participants for: (a) collagen content; (b) ultrasound‐based muscle thickness; (c) distribution of capillaries per myofibre; (d) myofibres with centrally located nuclei; (e) lipid droplets per area; (f) average capillaries in contact per myofibre; (g) satellite cells per 100 myofibres; (h) myofibre cross‐sectional area; (i) myofibre type‐specific cross‐sectional area; and (j) distribution. Each dot represents data from a single patient.

### 3.3 BoNT‐A

Capillary contacts per myofibre were positively correlated with the number of BoNT‐A injections (*P* = 0.04; Table 3). No other outcomes were statistically significant for the number of BoNT‐A injections, although the proportion of co‐expressing type IIa/IIx myofibres tended to decrease with the number of injections (*r*_s_ = −0.52, *P* = 0.06).

### 3.4 SDR

No outcomes were statistically significant for SDR, although the proportions of type IIa myofibres (*r*_rb_ = 0.89, *P* = 0.05) and collagen (*r*_rb_ = 1.00, *P* = 0.06) tended to be lower in the SDR group compared with the no‐SDR group (Table 4).

## 4 DISCUSSION

This exploratory study quantified variability in medial gastrocnemius properties by age, ambulatory status and spasticity treatment (BoNT‐A and SDR) in young people with CP. Its primary contribution is descriptive and hypothesis‐generating. The significant differences for each of the four independent variables suggest potentially meaningful differences, many of which align with previous prospective studies (Alexander et al., 2018; Barber et al., 2013; De Beukelaer et al., 2023, 2022; Deschrevel et al., 2024; Lambrechts et al., 2025; Peeters et al., 2022; Verreydt et al., 2025; Williams et al., 2013) and should be explored further in larger confirmatory studies.

### 4.1 Age

Our findings, although preliminary, found several medium or large effect sizes across the studied age range of 6–17 years, underscoring the importance of controlling for age when evaluating skeletal muscle. Myofibre CSA was positively correlated with age, which is consistent with growth trajectories in ambulatory children with CP (De Beukelaer et al., 2023), despite diminished growth potential compared with neurotypical peers (Barrett & Lichtwark, 2010; Eek & Beckung, 2008). The unexpected large, positive association of myofibres with centrally located nuclei and age might reflect heightened remodelling and repair during adolescence in CP muscle or it could reflect treatment effects, because the probability of having received systemic or gastrocnemius‐specific treatment generally increases as children with CP age. Central nuclei can be visualized during normal repair and maturation of myofibres (Folker & Baylies, 2013), but they can also indicate regeneration after damage (Roth et al., 2000). Replication by others is necessary to confirm this new finding, or exploration of covariates that might explain this observation.

Individuals with CP often experience greater muscle tightness or worsening joint contractures with age (Cloodt et al., 2021). Collagen is believed to contribute to muscle tightness or stiffness and perceived ease of movement. We did not find evidence of differences in collagen content across the childhood age range studied in this exploratory study, although studies inclusive of adults might reveal different trends. Still, absolute values of collagen were high compared with neurotypical peers, where a 1.5‐ to 3‐fold increase in collagen is common (Edman et al., 2025). Neurotypical children show increased capillarization over childhood (Andries et al., 2024), whereas we did not observe a correlation with age. The combined trend of fewer oxidative myofibres and no increase in capillaries with age might have an additive effect of fatigue, which should be explored in future studies.

### 4.2 Ambulatory status

Little is known about histological or biochemical differences between ambulatory and non‐ambulatory individuals with CP. Unsurprisingly, non‐ambulatory individuals had smaller muscles compared with ambulatory individuals, as measured by myofibre CSA and muscle thickness (borderline statistically significant), which concurs with others (Barrett & Lichtwark, 2010; Schless et al., 2019). The trend towards fewer satellite cells within the non‐ambulatory group might indicate a decreased potential or need for myofibre hypertrophy and repair. Kahn et al. (2023) found no difference by ambulatory status, but they did not hold the muscle source constant and also had small sample sizes. Larger, well‐controlled studies are needed to determine the robustness of this new finding and whether satellite cells or related pathways can be manipulated to improve muscle health in non‐ambulatory individuals.

### 4.3 Botulinum toxin

Although other cross‐sectional and longitudinal studies have noted causal effects or positive correlations between BoNT‐A injections and collagen and lipids (or their surrogate measures, such as ultrasound echo‐intensity) (De Beukelaer et al., 2022; Multani et al., 2019; Schless et al., 2019; Uzun Akkaya et al., 2025), we did not observe statistically significant correlations, although the direction of the relationship aligns with the literature. Additionally, myofibre type distributions should be explored further because there was a trend towards fewer co‐expressing type IIa/IIx myofibres as the number of BoNT‐A injections increased. Differences in myofibre types might affect the ease of certain physical activities, such as fatigue during walking, that stress the various energy systems differently. Other preliminary findings herein suggest a more favourable or null difference for BoNT‐A. Capillary contact per myofibre was positively associated with the number of injections. This finding agrees with the majority of CP and animal studies that show angiogenic effects of BoNT‐A (Deschrevel et al., 2024; Friden et al., 2022; Goldberg et al., 2023) but not all (Uzun Akkaya et al., 2025). Myofibre CSA did not differ by the number of BoNT‐A injections. Although timescales and measurement methods differ, another CP study reported a 14% decrease in CSA 3 months post‐BoNT‐A and recovery by 6 months, which was driven primarily by type IIa hypertrophy (Deschrevel et al., 2024). Given that repeat injection intervals of <12 months are common and preliminary data suggesting muscle growth in BoNT‐A‐naive children is more affected in comparison to those with a history of BoNT‐A (Lambrechts et al., 2025), real‐world effectiveness studies are needed to outline these nuances and address other study limitations to inform clinical recommendations regarding BoNT‐A.

### 4.4 SDR

To our knowledge, this is the first biopsy study, other than a single case study (Raymond‐Pope et al., 2023), to begin to explore whether muscle properties vary between individuals who underwent SDR versus those who did not. Wide effect size confidence intervals reflect small sample sizes, but some outcomes had non‐overlapping or minimally overlapping distributions, which suggest potential group differences worth exploring further. For instance, the SDR group trended towards lower collagen content compared with the no‐SDR group. Reduced collagen in SDR muscle concurs with the prior case report (Raymond‐Pope et al., 2023) and might provide a biological explanation for the reduced tightness and ease of movement that patients report anecdotally. Lower collagen might be mediated through lower spasticity or resting tone, fewer BoNT‐A injections that might otherwise increase collagen, or other mechanisms. Whether this persists into adulthood remains unknown. The trend towards fewer type IIa myofibres in the SDR group is similar to the prior single case report (Raymond‐Pope et al., 2023), suggesting that endurance activities might be easier but that power‐based activities might be more challenging. The SDR and no‐SDR groups might also have had different baseline characteristics, which should be explored.

### 4.5 Limitations

The main study limitations are the cross‐sectional nature, various prior treatments, small sample size and lack of neurotypical control data, given that ethical considerations prevented acquisition of muscle biopsies in neurotypical children. Owing to these limitations, causality cannot be inferred. Exploring potential age effects mixed with various treatment histories prevents distinguishing age from transient or long‐term treatment effects (or surgical vs. rehabilitation or ancillary treatment effects). Furthermore, muscle size was not normalized by body size. Future studies should explore sex differences, although they are expected to be minimal in young children (Deschrevel et al., 2024, 2025).

## 5 CONCLUSION

In conclusion, the results from this exploratory study of CP muscle suggest that there might be systematic differences in gastrocnemius properties by age and ambulatory status, in addition to spasticity treatment history (BoNT‐A or SDR). These findings highlight the potential relevance of these patient factors and treatment when describing and interpreting muscle biopsy data in children with CP. Larger, longitudinal confirmatory studies are needed to evaluate more definitively the short‐ and long‐term effects of treatment and non‐modifiable patient factors (age and ambulatory status) to generate evidence that can be shared with patients and their families.

## AUTHOR CONTRIBUTIONS

Ultrasounds and biopsies were gathered at Gillette Children's, 200 University Avene E, St. Paul, MN, USA. All biopsies were analysed at Dr Greising's Skeletal Muscle Plasticity and Regeneration Laboratory, Minneapolis, MN, USA. Elizabeth R. Boyer: Study conceptualization, study design, data analysis, statistical analyses, data interpretation, manuscript preparation and revisions and funding procurement. Christiana J. Raymond‐Pope: Study conceptualization, study design, data acquisition, data analysis, data interpretation, critical review of manuscript and funding procurement. Daniel B. Hoffman: Data acquisition, data analysis, data interpretation and critical review of manuscript. Marshall H. Taniguchi: Study conceptualization, study design, data acquisition, data interpretation, critical review of manuscript and funding procurement. Tom F. Novacheck: Study conceptualization, study design, data acquisition, data interpretation, critical review of manuscript and funding procurement. Braydon A. Crum: Data acquisition, data analysis, data interpretation and critical review of manuscript. Peter D. Kim: Data interpretation and critical review of manuscript. Sarah M. Greising: Study conceptualization, study design, data acquisition, data analysis, data interpretation, critical review of manuscript and funding procurement. All authors approved the final version of the manuscript and agree to be accountable for all aspects of the work in ensuring that questions related to the accuracy or integrity of any part of the work are appropriately investigated and resolved. All persons designated as authors qualify for authorship, and all those who qualify for authorship are listed.

## CONFLICT OF INTEREST

The authors declare no conficts of interest.

## GENERATIVE AI STATEMENT

Generative AI (Claude Sonnet v.4.6) was used to aid in selecting appropriate and alternative statistical analyses, writing R code and troubleshooting R code. The first author verified all primary sources and accuracy.

## Data Availability

The data that support the findings of this study are available from the corresponding author upon reasonable request.

## 1

**TABLE A1:** Other prior treatments, detailed ages at gastrocnemius treatment, and current treatment at time of biopsy.

ID	Side	Other prior treatments (not specific to biopsied gastrocnemius)	Detailed ages at prior gastrocnemius treatment	Other prior gastroc or global treatment	Current treatment
**1**	R	1) Multiple BoNT‐A to multiple bilateral upper extremity (UE) muscles, 2) Multiple BoNT‐A to bilateral rectus femoris, 3) Multiple phenol to bilateral obturator nerve	**Phenol**: 5 y 11 mo, 6 y 2 mo, 6 y 5 mo, 6 y 8 mo, 6 y 11 mo, 7 y 2 mo, 7 y 8 mo, 7 y 11 mo, 8 y 2 mo, 8 y 5 mo, 8 y 8 mo, 8 y 11 mo, 9 y 4 mo, 9 y 7 mo, 9 y 10 mo, 10 y 1 mo, 10 y 5 mo, 10 y 8 mo, 11 y 0 mo, 11 y 2 mo, 11 y 6 mo, 11 y 9 mo	Phenol	Phenol to bilateral gastrocnemius and obturator nerves; BoNT‐A to bilateral rectus femoris
**2**	R	1) Multiple BoNT‐A to left hamstrings & gastroc, 2) BoNT‐A to left adductors, 3) phenol bilateral obturator nerve	**BoNT‐A**: 3 y 11 mo, 4 y 4 mo, 5 y 0 mo, 5 y 6 mo, 6 y 4 mo	N/A	SDR
**3**	L	BoNT‐A bilateral hamstrings	**Shunt**: 0 y 2 mo **Detethering**: 6 y 0 mo	VP shunt; cord detethering	SEMLS
**4**	R	1) Multiple BoNT‐A to bilateral hamstrings & posterior tibialis, 2) alcohol to bilateral obturator & sciatic	N/A	N/A	SEMLS
**5**	L	1) Left adductor lengthening, 2) Multiple phenol to bilateral obturator nerve, 3) Multiple phenol bilateral musculocutaneous nerve, 4) multiple BoNT‐A bilateral hamstrings, 5) multiple BoNT‐A UE, 6) bilateral femoral derotation osteotomy	**BoNT‐A**: 6 y 6 mo, 7 y 0 mo **Strayer lengthening**: 7 y 4 mo	Strayer gastroc lengthening	Phenol to bilateral musculocutaneous nerve
**6**	R	1) Right ankle cast, 2) Left femoral epiphysiodesis	**BoNT‐A**: 4 y 6 mo	N/A	SEMLS
**7**	R	N/A	**BoNT‐A**: 3 y 0 mo, 3 y 8 mo, 4 y 5 mo, 4 y 8 mo, 5 y 2 mo, 5 y 6 mo, 5 y 10 mo, 6 y 2 mo, 6 y 7 mo, 7 y 0 mo, 7 y 4 mo, 7 y 8 mo, 9 y 1 mo, 9 y 6 mo, 12 y 1 mo, 13 y 4 mo, 13 y 8 mo; **Right ankle serial casting**: 3 y 0 mo, 7 y 0 mo, 9 y 1 mo, 9 y 6 mo, 12 y 1 mo, 13 y 4 mo, 13 y 8 mo	Multiple right ankle serial casting	SEMLS
**8**	R	Bilateral flexor lengthening, posterior compartment and peritalar joint release for club foot repair, cuboid osteotomy & split anterior tibial tendon transfer	**Achilles lengthening**: 1 y 1 mo, 2 y 2 mo	Bilateral Achilles lengthening	BoNT‐A to bilateral flexor digitorum longus and flexor digitorum brevis
**9**	L	1) Multiple BoNT‐A to multiple bilateral UE muscles, 2) Multiple BoNT‐A to bilateral hamstrings and soleus	**BoNT‐A**: 11 y 7 mo, 12 y 0 mo, 12 y 4 mo, 12 y 7 mo, 13 y 1 mo, 13 y 4 mo, 13 y 7 mo, 14 y 0 mo, 14 y 5 mo **Shunt**: 0 y 2 mo, **Shunt revision**: 9 y 10 mo, 13 y 10 mo	VP shunt & revision	SDR
**10**	L	1) BoNT‐A bilateral hip adductors & hamstrings, 2) bilateral foot reconstruction, Left tibial derotation osteotomy	**BoNT‐A**: 4 y 4 mo **Baumann recession**: 7 y 8mo	Baumann gastroc recession	SEMLS
**11**	R	1) Multiple BoNT‐A to bilateral hamstrings, 2) Multiple phenol to bilateral obturator nerve, 3) BoNT‐A bilateral rectus femoris, iliopsoas	N/A	N/A	SEMLS
**12**	L	1) Multiple bilateral phenol to obturator nerve, 2) Multiple BoNT‐A bilateral hamstrings & adductors, 3) Bilateral varus femoral derotation osteotomy & repeat varus femoral derotation osteotomy, 4) Bilateral calcaneus osteotomy	**BoNT‐A**: 4 y 11 mo **Strayer lengthening**: 7 y 6 mo	Strayer gastroc lengthening	BoNT‐A to bilateral medial hamstrings
**13**	R	1) Bilateral varus femoral derotation osteotomy, 2) Bilateral San Diego pelvic osteotomy	N/A	N/A	SEMLS
**14**	R	1) Bilateral Albee pelvic osteotomy, 2) bilateral psoas release, 3) bilateral varus femoral derotation osteotomy, 4) Multiple BoNT‐A various UE muscles, 5) Multiple BoNT‐A hamstrings & adductors	N/A	N/A	SEMLS
**15**	R	1) Multiple bilateral phenol to obturator nerve, 2) Multiple BoNT‐A bilateral iliopsoas, hamstrings, rectus femoris, adductors, extensor hallucis longus, 3) Bilateral distal femoral varus osteotomy, 4) Bilateral Pemberton pelvic osteotomy	**BoNT‐A**: 5 y 0 mo, 5 y 2 mo, 5 y 8 mo, 6 y 0 mo	N/A	BoNT‐A to multiple upper extremity muscles
**16**	R	1) Bilateral adductor lengthening, 2) Bilateral psoas release, 3) Multiple bilateral phenol obturator nerve, 4) Bilateral open reduction hip and capsulotomy, 5) Multiple BoNT‐A bilateral iliopsoas, hamstrings, 6) BoNT‐A right anterior tibialis, peroneus brevis & longus	**BoNT‐A**: 8 y 1 mo, 9 y 4 mo	N/A	BoNT‐A to bilateral psoas, bilateral hamstrings, right extensor hallucis longus; Phenol to bilateral obturators bilateral distal anterior femur hemiephiphysiodesis
